# Single-fiber co-registered Raman-OCT technique with deep learning enhances *in vivo* oral tissue assessment

**DOI:** 10.1117/1.BIOS.3.3.035004

**Published:** 2026-08-27

**Authors:** Chang Liu, Bin He, Kan Lin, Chi Shu, Wei Zheng, Zhiwei Huang

**Affiliations:** a National University of Singapore, College of Design and Engineering, Department of Biomedical Engineering, Optical Bioimaging Laboratory, Singapore; b National University of Singapore (Suzhou) Research Institute, Suzhou, China; c National University of Singapore Guangzhou Research Translation and Innovation Institute, Guangzhou, China; d National University of Singapore, NUS Graduate School for Integrative Sciences and Engineering Programme (ISEP), Singapore

**Keywords:** fiberoptic Raman spectroscopy and optical coherence tomography, *in vivo* oral tissue analysis, deep learning

## Abstract

Conventional oral cancer diagnosis relies on white light imaging and tissue biopsy that fail to capture critical microstructural and biomolecular information, thereby limiting early detection of precancerous and malignant lesions. To address these challenges, we present a unique coaxial and co-registered morpho-chemical imaging platform that integrates Raman spectroscopy (RS) and optical coherence tomography (OCT) within a compact, single-fiber probe. This integration uniquely enables video-rate OCT imaging together with sub-second Raman acquisition, providing spatially aligned microstructural and biochemical tissue information *in vivo*. Specifically, the single-fiber RS-OCT probe is constructed using a double-clad fiber (DCF) and gradient index (GRIN) lens architecture. The DCF’s single-mode core delivers and collects OCT light for high-resolution structural imaging, whereas its high-numerical-aperture multimode inner cladding coupled to the GRIN fiber lens facilitates efficient Raman excitation and collection along an identical optical axis, ensuring truly coaxial and co-registered tissue measurements. A free-space coupling scheme incorporating spatial filtering effectively suppresses both fiber-originated Raman background and DCF-induced OCT multipath artifacts, achieving artifact-free biochemical–microstructural integration within a compact intraoral probe. The resulting system achieves ∼100  dB OCT imaging sensitivity and acquires both fingerprint (880 to $1800\;{\mathrm{cm}}^{-1}$) and high-wavenumber (2800 to $3600\;{\mathrm{cm}}^{-1}$) tissue Raman spectra within sub-seconds. We validated the hybrid RS-OCT system through *in vivo* oral measurements across nine anatomically distinct intraoral sites in healthy volunteers, including challenging posterior tongue regions. To synergistically fuse the co-registered morpho-chemical datasets, we implemented a cross-modality deep learning framework. This model, integrating OCT patch-voting with a dual-region Raman branch (fingerprint and high wavenumber), achieved a superior overall classification accuracy of 92.38% across all nine sites. This represents a significant diagnostic improvement of 16.55% and 21.19% over Raman-only (75.83%) and OCT-only (71.19%) modalities, respectively. Notably, the RS-OCT cross-modality deep learning model attained 93.4% accuracy within high-risk tongue sub-regions. This work establishes the single-fiber-enabled hybrid RS-OCT technique as a promising tool for enhanced *in vivo* oral tissue diagnosis with strong potential for broader clinical translations.

Statement of Discovery This work exemplifies the convergence of advanced biomedical instrumentation and intelligent data-driven analysis to enable next-generation, point-of-care diagnostic solutions with clear translational potential. (1) First demonstration of a truly coaxial, single-fiber Raman–OCT probe for *in vivo* use; (2) seamless integration of structural and biochemical imaging at clinically relevant speeds; (3) deep learning–enabled cross-modality fusion that unlocks synergistic diagnostic performance.

## 1 Introduction

Early detection of oral malignancies remains a formidable clinical challenge, hindered by the subtle and often multifocal nature of dysplastic changes, compounded by the intricate anatomy of the oral cavity. The malignant transformation of the oral mucosa is not a singular event but a dynamic micro-evolution, wherein the structural disorganization of the epithelium, such as the loss of normal stratification, occurs concurrently with biochemical remodeling of the underlying stroma, including collagen degradation and shifts in cellular metabolism. Unlike many planar epithelial sites, the oral mucosa exhibits profound structural and biochemical heterogeneity, characterized by layered epithelia, a collagen-rich stroma, and variable vasculature.^1^^–^^4^ Diagnostic methods relying on a single contrast mechanism inherently fail to capture this complex interplay, resulting in significant ambiguity for accurate risk stratification.^3^ Consequently, a diagnostic strategy capable of jointly acquiring depth-resolved structural features alongside molecularly specific chemical signatures is paramount for enhancing *in vivo* characterization of early oral disease.

A hybrid probe combining Raman spectroscopy (RS) and optical coherence tomography (OCT) presents an inherently synergistic solution to decode this biological complexity. RS is a label-free vibrational technique exquisitely sensitive to biochemical fingerprints, including stromal collagen remodeling and variations in lipid and protein composition, and has been extensively investigated for tissue diagnosis.^5^^–^^13^ OCT, by contrast, detects backscattered light from refractive-index variations, providing micrometer-scale visualization of epithelial architecture and showing significant promise for identifying microstructural disruptions associated with neoplastic progression in the oral cavity.^14^^–^^18^ Despite this synergy, clinical translation has been impeded by significant technological bottlenecks. Dual-probe designs are inherently susceptible to motion-induced co-registration errors, compromising sampling of the same tissue site. Conversely, bulky free-space optics for beam combination impose severe form-factor constraints, creating anatomical blind spots that preclude access to clinically high-risk regions such as the tongue base.^19^^–^^25^

These limitations motivate the development of a co-registered, morpho-chemical RS-OCT platform with broad anatomical access, a goal that fundamentally necessitates a single-fiber design to minimize probe footprint and guarantee intrinsic co-registration. However, integrating both modalities within a single waveguide introduces a critical physical trade-off arising from their opposing optical requirements. Raman signal collection benefits from large-core, multimode fibers with high numerical aperture (NA) for efficient light capture, whereas OCT typically relies on single-mode, lower-NA delivery fibers to maintain beam quality and resolution. This dichotomy has motivated the use of a double-clad fiber (DCF) to provide coaxial dual channels. ^26^^–^^29^ However, implementing a DCF-based RS-OCT system confronts a fundamental design conflict. On the one hand, the Raman channel is highly susceptible to background signals generated spontaneously within the fiber glass.^26^ As depositing coating filters directly on the DCF tip is technically impractical, the fiber length must be strictly minimized to prevent this background from overwhelming the detector and suppressing the measurement’s dynamic range.^5^ On the other hand, DCF-based OCT inherently suffers from multipath artifacts.^27^ Conventional systems typically employ long fiber segments to delay these artifacts sufficiently so they fall outside the imaging range.^28^^–^^29^ Shortening the fiber to accommodate the Raman requirements, however, draws these artifacts closer to the true signal, inevitably degrading OCT image quality. These opposing constraints—the need for a short fiber to suppress Raman background and a long fiber to mitigate OCT artifacts—have, until now, hindered the development of truly compact RS-OCT probes and limited their application for co-registered structural and chemical measurements in the oral cavity.

In this study, we present an innovative single-fiber RS-OCT imaging platform that resolves these opposing constraints to enable truly coaxial, co-registered intraoral diagnostics. We introduce a unique optical architecture employing precision free-space coupling combined with a spatial filtering aperture. This design allows us to utilize the requisite short fiber for Raman background suppression while simultaneously mitigating OCT multipath artifacts. By overcoming this critical bottleneck, we successfully packaged the system into a handheld probe capable of accessing anatomically challenging regions, such as the posterior tongue. Furthermore, to fully exploit the rich, synergistic data, we implemented an attention-gated, multibranch deep learning framework that integrates OCT patch-voting with dual-range Raman inputs. Our *in vivo* validation demonstrates that this integrated approach yields high-fidelity, co-registered structural and chemical measurements, significantly enhancing tissue discrimination compared with either modality alone and establishing a viable pathway for real-time, comprehensive oral screening.

## 2 Results

### 2.1 Single-Fiber-Enabled RS-OCT System

**Fig. 1:**
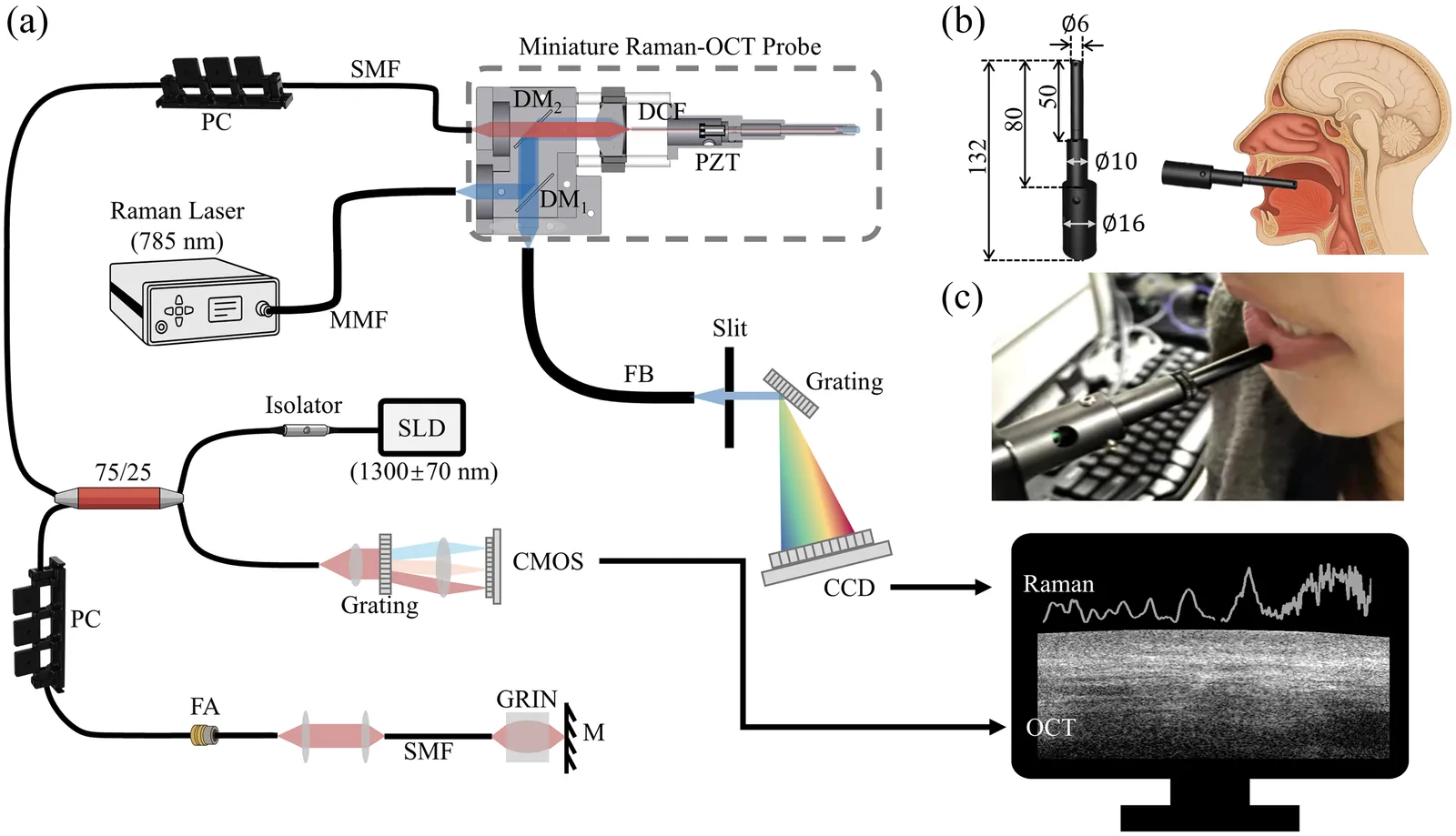
Single-fiber-based hybrid Raman spectroscopy–optical coherence tomography (RS–OCT) system for co-registered *in vivo* intraoral measurements. (a) Schematic of the RS–OCT system based on a single fiber. A 785-nm Raman laser is delivered and collected through the DCF inner cladding, whereas a 1300±70  nm SLD-based OCT subsystem is coupled to the single-mode core; ${\mathrm{DM}}_{1}$ and ${\mathrm{DM}}_{2}$ integrate and separate the RS and OCT paths, enabling co-axial and co-registered *in vivo* intraoral tissue measurements. PC, polarization controller; MMF, multimode fiber; FA, fiber attenuator; SMF, single-mode fiber; DCF, double-clad fiber; CCD, charge-coupled device; FB, fiber bundle; DM, dichroic mirror; PZT, piezoelectric transducer; GRIN, gradient-index lens; M, mirror; SLD, super-luminescent diode. (b) Design and dimensions of the miniature stepped DCF-based Raman–OCT probe. The probe has a 6-mm-diameter distal tip for access to confined intraoral regions, followed by 10-mm- and 16-mm-diameter sections for fiber alignment and mechanical packaging. The total probe-head length is 132 mm. All dimensions shown are in millimeters. (c) *In vivo* intraoral RS–OCT measurement using the miniature probe.

**Fig. 2:**
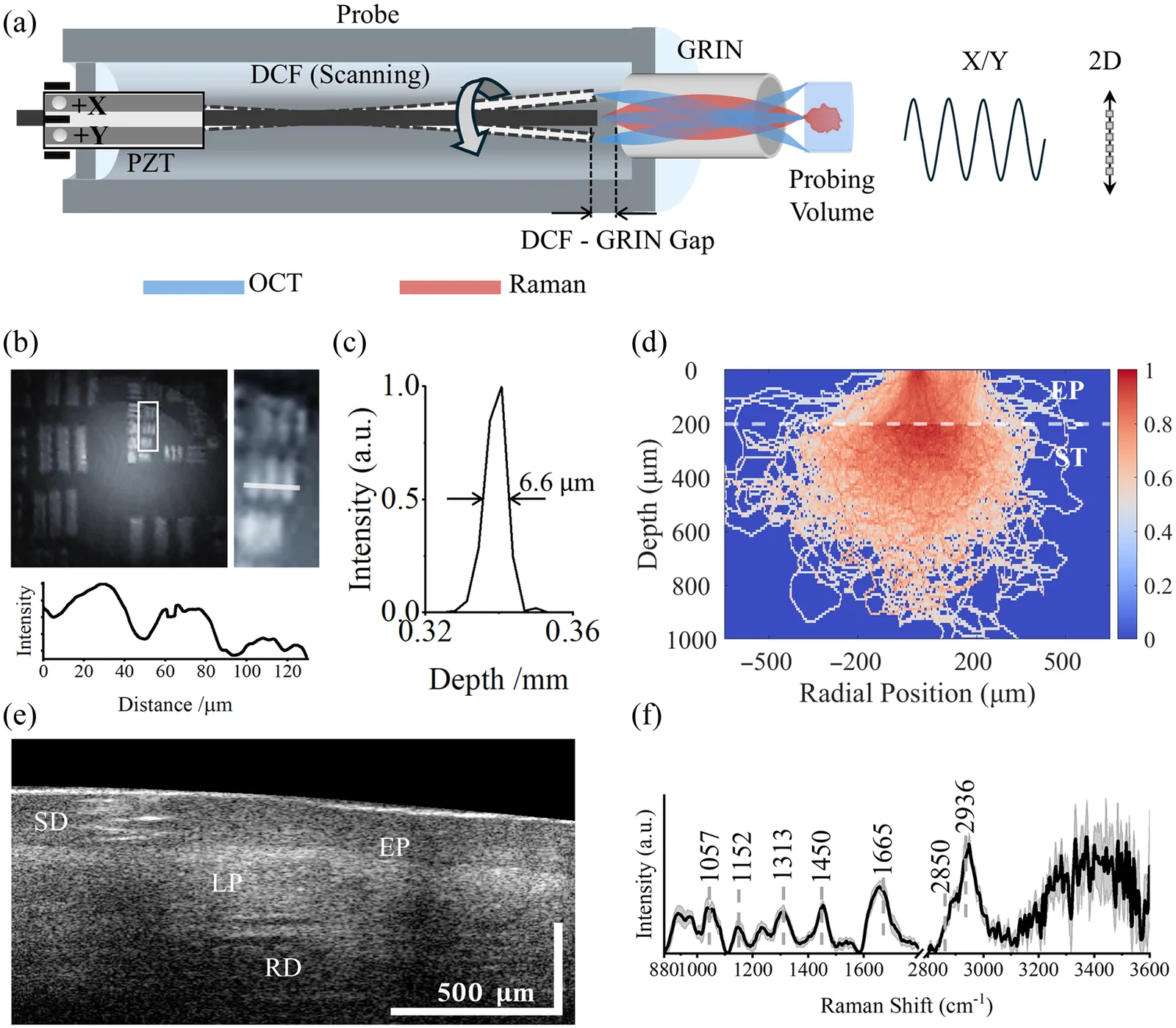
Probe design, system performance, and representative *in vivo* results of the single-fiber-based RS–OCT platform. (a) Schematic illustration of co-axial Raman (red) and OCT (blue) beam propagation through the DCF and GRIN, enabling co-registered detection at the tissue. (b) USAF resolution target and measured lateral intensity profile, demonstrating a lateral resolution of 22  μm. (c) Point-spread function (PSF) and corresponding axial resolution (6.6  μm in air, 3 dB). (d) Distribution of Raman photons in a two-layer tissue model (epithelium and stroma) collected by detector using Monte Carlo (MC) methods. The color bar represents the normalized intensity. EP, epithelium; ST, stroma. (e) Representative *in vivo* OCT image of a human fingertip, showing key anatomical layers: sweat duct (SD), EP, lamina propria (LP), and reticular dermis (RD). (f) Representative *in vivo* Raman spectrum acquired from the fingertip, with major Raman bands labeled.

The single-fiber-enabled RS-OCT system [Fig. 1(a)] consists of three parts: (i) The excitation and signal collection module for RS. A 785-nm laser provides Raman excitation, and the backscattered Raman signal is routed into a fiber bundle and delivered to the charge-coupled device (CCD) spectrograph for spectral acquisition. (ii) The illumination and detection design for OCT imaging module. A 1300±70  nm super-luminescent diode (SLD)-based light source operating at a 48-kHz A-line rate is utilized for OCT subsystem, and a 75/25 coupler is used to ensure efficient light distribution between the sample and reference arms. Polarization control is implemented to manage polarization effects, whereas a dispersion-matched reference arm is used to minimize distortions from dispersion in the optical fibers. Returning beams from two arms are then combined and directed to the OCT spectrometer for signal acquisition. (iii) A custom-designed handheld probe. A pair of dichroic mirrors integrate and separate the RS and OCT paths, enabling co-axial light delivery to the front of probe and co-registered intraoral tissue measurements. The clinically compatible probe has a 6-mm-diameter distal front end for access to confined intraoral regions, whereas the stepped 10-mm- and 16-mm-diameter sections provide mechanical support for fiber alignment and probe packaging; the total rigid probe-head length is 132 mm [Fig. 1(b)]. Co-registered *in vivo* OCT structural and Raman spectral data can be acquired using this probe at multiple intraoral sites [Fig. 1(c)]. Inside the handheld probe, Raman (red) and OCT (blue) beams are delivered and collected through a DCF, with the OCT beam guided in the central core and the Raman excitation and emission coupled through the inner cladding [Fig. 2(a)].

To guarantee efficient signal transmission and ensure that the OCT beam is strictly confined to the fundamental mode (FM, ${\mathrm{LP}}_{01}$) within the core, precise control of the proximal coupling is essential. Specifically, we employed a mode-stripping strategy by removing the DCF coating, applying index-matching oil, and inducing macro bending. Experimentally, we achieved a total OCT coupling efficiency of 83.19%, with a confirmed FM power fraction of 92.21%. This high-purity core transmission demonstrates effective suppression of initial higher-order modes (HOMs, e.g., modes ${\mathrm{LP}}_{11}$, ${\mathrm{LP}}_{21}$, ${\mathrm{LP}}_{02}$, ${\mathrm{LP}}_{12}$, and ${\mathrm{LP}}_{22}$), aligning well with numerical simulations (for details, refer to Sec. S1 in the Supplementary Material). Simultaneously, the co-aligned Raman channel attained an inner-cladding coupling efficiency of ∼77.57%, delivering sufficient excitation power to the probe tip. A short gradient index (GRIN) lens is used to balance the numerical aperture and ensure co-registration. A 1.2-mm DCF-GRIN distance is chosen to balance these parameters, resulting in a working distance of 0.6 mm, NA of 0.5 for RS, and NA of 0.15 for OCT (for details, refer to Sec. S2 in the Supplementary Material). A piezoelectric tube mounted at the proximal end of the DCF drives lateral beam scanning within the compact 6-mm-diameter probe head while maintaining strict co-alignment of the Raman and OCT sampling regions.

The imaging performance of the probe was quantified using standard optical targets under the circular scanning mode. As shown in Fig. 2(b), the OCT beam could clearly resolve elements 4-4 on USAF resolution target, and the corresponding lateral intensity profile confirmed a lateral resolution of 22  μm. The axial point-spread function (PSF) measured in air [Fig. 2(c)] exhibited a full-width at half-maximum of ∼6.6  μm, consistent with the spectral bandwidth of the OCT engine and demonstrating high depth-resolution suitable for resolving thin epithelial layers. The OCT subsystem sensitivity achieves ∼100  dB. To evaluate Raman collection efficiency and sampling depth, we performed Monte Carlo simulations of photon transport in a two-layer tissue model consisting of epithelium (EP) overlying stroma (ST) [Fig. 2(d); for details, refer to Sec. S3 in the Supplementary Material].^26^^–^^28^ The Raman signal interrogates tissue laterally over a radius of ∼500  μm and vertically to a depth of ∼1000  μm, forming the primary contribution to the detected spectrum. The simulated axial interrogation range was further supported by experimental characterization of the axial Raman response (Fig. S1 in the Supplementary Material). This Raman sampling volume lies within the OCT scan field, such that the two modalities interrogate overlapping tissue volumes at the same measurement site. This spatial overlap along the shared optical axis defines the co-registration in this work.

Representative *in vivo* measurements further illustrate the capability of the DCF–GRIN RS–OCT platform. Figure 2(e) shows an OCT B-scan of a human fingertip acquired with the probe, where key anatomical structures, including the sweat duct (SD), EP, lamina propria (LP), and reticular dermis (RD), are clearly visualized with sufficient contrast and depth penetration. Co-registered Raman spectra [Fig. 2(f)] exhibit prominent bands associated with major biochemical constituents, such as $1057\;{\mathrm{cm}}^{-1}$ (lipids), $1152\;{\mathrm{cm}}^{-1}$ (C─C stretching in lipids/carotenoids), $1313\;{\mathrm{cm}}^{-1}$ (CH deformation in proteins/collagen), $1450\;{\mathrm{cm}}^{-1}$ (${\mathrm{CH}}_{2}/{\mathrm{CH}}_{3}$ bending of lipids and proteins), $1665\;{\mathrm{cm}}^{-1}$ (amide I, C═O stretching of proteins), and 2850 and $2935\;{\mathrm{cm}}^{-1}$ (${\mathrm{CH}}_{2}/{\mathrm{CH}}_{3}$ stretching in lipids and proteins). Collectively, these results confirm that the proposed DCF-GRIN probe delivers high-resolution structural imaging and chemically specific Raman readouts from a common, well-defined sampling volume, forming the basis for co-registered *in vivo* RS–OCT measurements.

### 2.2 Multipath Artifact Suppression

**Fig. 3:**
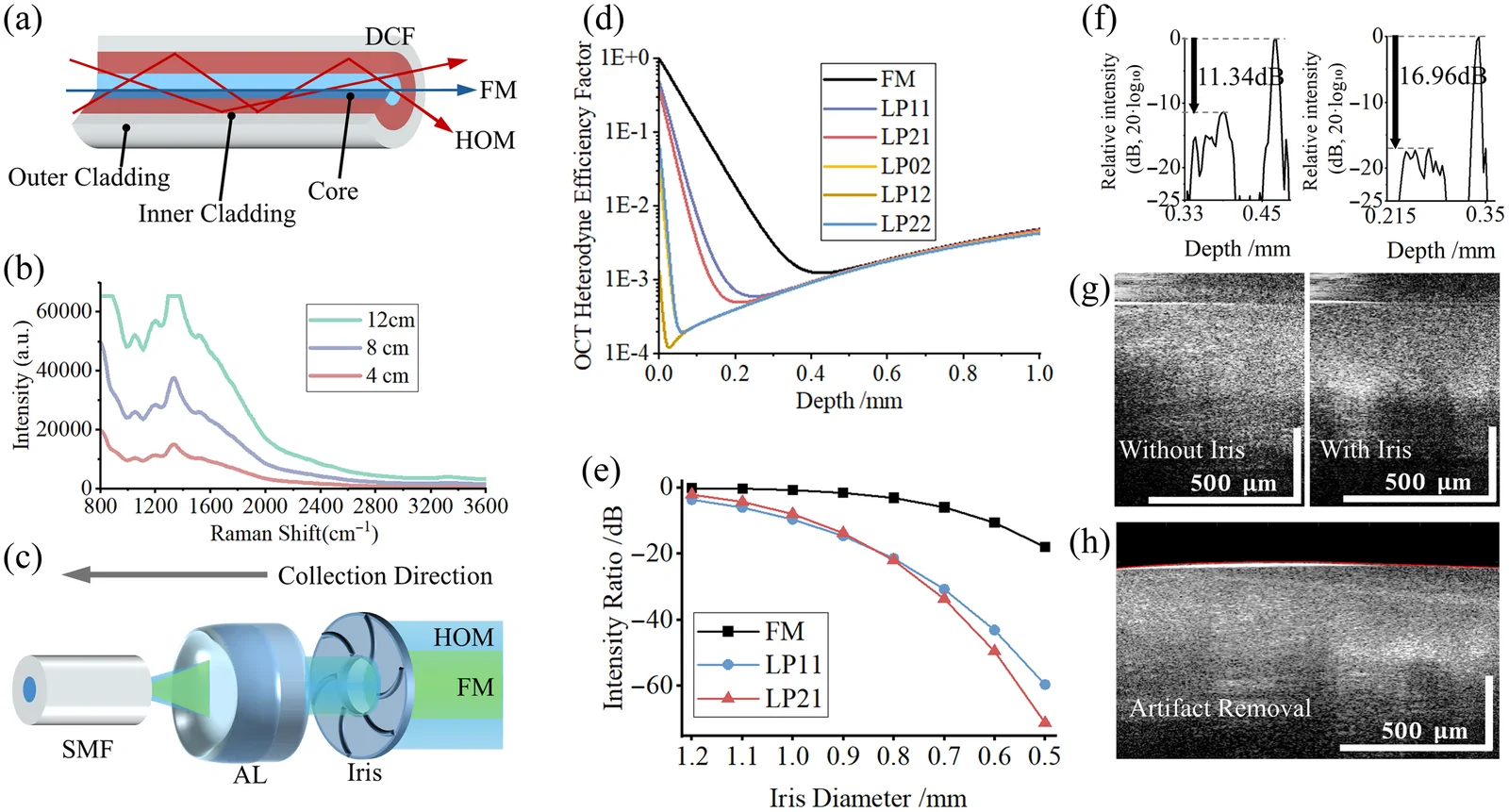
Generation, effect, and suppression of higher-order modes (HOMs). (a) Schematic of the DCF structure and propagation of the fundamental mode (FM) in the core and HOMs in the inner cladding. (b) Measured fluorescence background as a function of DCF length, showing rapid background growth that limits the usable fiber length for RS–OCT. (c) Schematic of the spatial filtering aperture placed in the relay optics to block peripheral rays and preferentially transmit the central FM component. (d) Depth-dependent OCT heterodyne efficiency factors for the fundamental mode (FM) and five representative higher-order modes (HOMs, e.g., ${\mathrm{LP}}_{11}$, ${\mathrm{LP}}_{21}$, ${\mathrm{LP}}_{02}$, ${\mathrm{LP}}_{12}$ and ${\mathrm{LP}}_{22}$), quantifying the relative contribution of HOM crosstalk to the OCT signal. (e) Calculated intensity ratios of FM, ${\mathrm{LP}}_{11}$, and ${\mathrm{LP}}_{21}$ versus iris diameter. An iris diameter of 0.9 mm (dashed line) is chosen as a trade-off, yielding <2  dB loss for the FM and >5  dB attenuation for HOMs. (f) Experimental OCT PSFs without (left) and with (right) the iris, where the HOMs peak is ∼11.34 and ∼16.96  dB below the FM, respectively. (g) *In vivo* lip OCT B-scan images without iris (left) and with iris (right). (h) *In vivo* lip OCT B-scan image after post-processing, the red line represents the identified surface.

DCF plays a crucial role in the system by simultaneously accommodating the conflicting modal requirements of the two modalities and providing naturally co-axial, co-registered channels for RS and OCT. However, it also introduces multipath artifacts in the OCT images. Figure 3(a) illustrates the propagation of the FM in the DCF core and HOMs in the inner cladding. In principle, the HOMs are used only for Raman excitation and collection, whereas the OCT subsystem should exclusively detect the FM in the core. Forward coupling yields a high FM ratio and low HOMs excitation through precise control, but backward large-angle signal and residual mode coupling allows core-like HOMs to be coupled back into the OCT detection path, generating multipath artifacts. The depth offset between the FM and these core-like HOMs is proportional to the DCF length and the modal group index difference. Conventional DCF-based OCT systems therefore often employ a long DCF segment so that the multipath artifacts fall outside the imaging range and do not overlap with the true image. In a DCF-based RS–OCT system, this strategy is not feasible because the fiber-generated Raman and fluorescence background increases with fiber length [Fig. 3(b)]. Under typical *in vivo* acquisition conditions with a 0.5-s exposure time, a DCF length of ∼12  cm is sufficient to saturate the CCD due to this background, severely compressing the usable dynamic range for Raman detection. On the other hand, a shortened DCF segment causes the multipath artifacts to overlap with the true OCT image, making their suppression critically important.

To address this issue, we adopted a spatial filtering aperture (iris diaphragm) placed in the relay optics [Fig. 3(c)] to block peripheral rays and preferentially transmit the central portion of the beam, thereby reducing the contribution of HOMs while largely preserving the FM. To quantitatively characterize the intensities of different HOMs, we first simulated the depth-dependent heterodyne efficiency of the FM and five representative HOMs using an extended Huygens-Fresnel model [Fig. 3(d); details in Sec. S4 in the Supplementary Material], yielding the HOMs’ raw back-scattered intensity ratios relative to the FM. Combined with the coupling efficiency into the collection fiber (Sec. S5.1 in the Supplementary Material), the overall intensity ratios of $LP_{11}$ and $LP_{21}$ to the FM are 24.25% and 21.16%, respectively, identifying them as the dominant contributors to multipath artifacts that require suppression. The aperture reduces the effective beam diameter and numerical aperture (NA) of HOMs, causing power loss and spot-size broadening of HOMs, which correspond to reductions in both their intensity and coupling efficiency into the single-mode collection fiber. Importantly, the iris would not affect OCT excitation or the Raman excitation/collection channel. Figure 3(e) summarizes the FM and dominant HOMs intensity ratios as a function of aperture diameter. As the aperture diameter decreases, the HOMs are strongly attenuated while the FM experiences only modest loss. We therefore selected an iris diameter of 0.9 mm, for which the FM attenuation is <2  dB, whereas the attenuation of ${\mathrm{LP}}_{11}$ and ${\mathrm{LP}}_{21}$ exceeds 5 dB. Experimentally measured PSFs with and without the spatial filtering aperture are compared in Fig. 3(f). The relative HOMs peak intensity is ∼−11.34  dB below the FM without the aperture and ∼−16.96  dB below the FM with the aperture, in good agreement with the simulations. *In vivo* finger OCT images in Fig. 3(g) further demonstrate that the aperture markedly suppresses the ghost interface arising from multipath artifacts and separates it from the true tissue surface. As shown in Fig. 3(h), to further minimize the impact of residual multipath artifacts, we implemented a U-Net-based post-processing step that detects the tissue surface and removes signals above it, yielding artifact-reduced OCT images.

### 2.3 Intraoral *In Vivo* RS-OCT Measurements

**Fig. 4:**
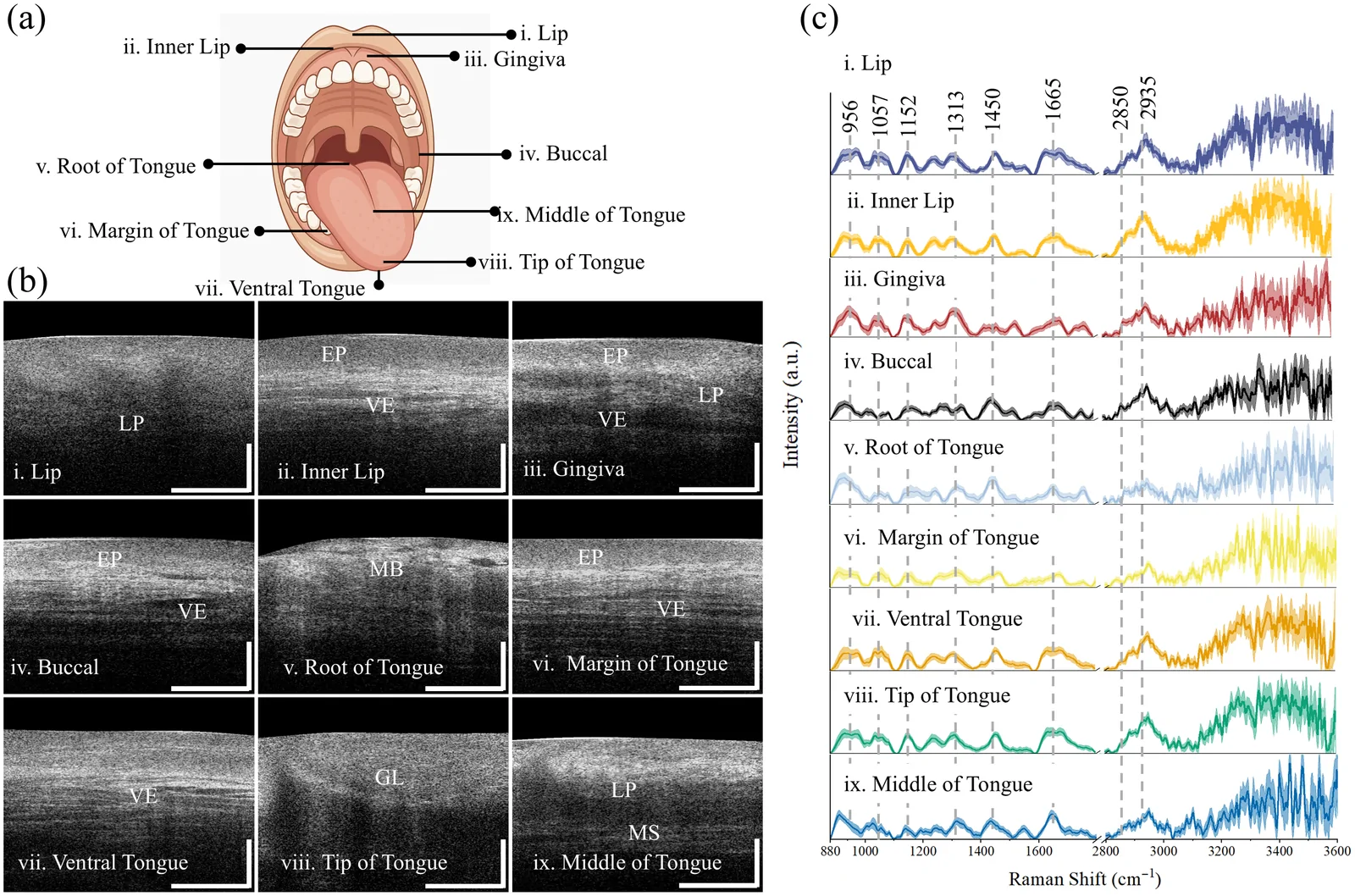
*In vivo* intraoral co-registered RS–OCT measurements at different oral sites. (a) Schematic illustration of the nine measurement locations: (i) lip, (ii) inner lip, (iii) gingiva, (iv) buccal mucosa, (v) root of tongue, (vi) margin of tongue, (vii) ventral tongue, (viii) tip of tongue, and (ix) middle of tongue. (b) Representative OCT B-scan images acquired at each site, revealing the layered microstructure of the oral mucosa. EP, epithelium; VE, blood vessels; LP, lamina propria; MB, muscle bundle; GL, gland; MS, muscle. The scale bar is 250  μm (lateral and axial). (c) Corresponding representative Raman spectra from the nine locations, showing site-dependent biochemical contrast. Major Raman bands include $∼956\;{\mathrm{cm}}^{-1}$ (phosphate/collagen), $1152\;{\mathrm{cm}}^{-1}$ (C─C stretching in lipids/carotenoids), $1313\;{\mathrm{cm}}^{-1}$ (CH deformation in proteins/collagen), $1450\;{\mathrm{cm}}^{-1}$ (${\mathrm{CH}}_{2}/{\mathrm{CH}}_{3}$ bending of lipids and proteins), $1665\;{\mathrm{cm}}^{-1}$ (amide I, C═O stretching of proteins), and 2850 and $2935\;{\mathrm{cm}}^{-1}$ (${\mathrm{CH}}_{2}/{\mathrm{CH}}_{3}$ stretching in lipids and proteins).

*In vivo* intraoral RS–OCT measurements were performed in 15 healthy volunteers at nine anatomically distinct oral sites [lip, inner lip, gingiva, buccal mucosa, root, margin, ventral, tip and middle of the tongue; as shown in Fig. 4(a)]. These regions were selected based on their reported relevance to oral malignancies,^2^^,^^30^^,^^31^ enabling the establishment of region-specific normal reference profiles. At each site, OCT B-scans were acquired at an A-line rate of 48 kHz, followed by a co-registered Raman spectrum from the same field of view using a 0.5-s integration time. The representative OCT images in Fig. 4(b) reveal the characteristic layered microstructure of the oral mucosa, with a thin to moderately thick EP overlying the more heterogeneous LP. Depending on location, additional subsurface features can be identified, including mucosal boundaries and papillary structures at the tongue root, minor salivary glands (GL) in the ventral tongue and tongue tip, and underlying muscle (MS) in the middle of the tongue. Variations in epithelial thickness, surface contour, and stromal scattering across the nine sites highlight the pronounced anatomical heterogeneity of the oral cavity.

The corresponding Raman spectra in Fig. 4(c) exhibit reproducible, region-dependent biochemical variations, with mean intra-region and inter-region spectral Pearson correlation coefficients of 0.80 and 0.47, respectively. All locations show prominent bands near $956\;{\mathrm{cm}}^{-1}$ (phosphate/collagen), $1152\;{\mathrm{cm}}^{-1}$ (C─C stretching in lipids and carotenoids), $1313\;{\mathrm{cm}}^{-1}$ (CH deformation in proteins/collagen), $1450\;{\mathrm{cm}}^{-1}$ (${\mathrm{CH}}_{2}/{\mathrm{CH}}_{3}$ bending of lipids and proteins), $1665\;{\mathrm{cm}}^{-1}$ (amide I of proteins), and 2850 to $2935\;{\mathrm{cm}}^{-1}$ (${\mathrm{CH}}_{2}/{\mathrm{CH}}_{3}$ stretching in lipids and proteins), consistent with the mixed epithelial–stromal composition of oral tissues.^6^ However, the relative amplitudes of these bands differ between sites: regions with thicker keratinized epithelium or collagen-rich stroma (e.g., gingiva, margin of tongue) show enhanced protein and collagen–related peaks around 1313 and $1665\;{\mathrm{cm}}^{-1}$, whereas sites such as the lip and buccal mucosa exhibit comparatively stronger lipid-associated CH stretching bands at 2850 and $2935\;{\mathrm{cm}}^{-1}$. These combined structural and spectroscopic contrasts provide a rich, co-registered dataset for subsequent anatomical and tissue-type classification of intraoral sites.

### 2.4 Cross-Modality RS-OCT Classifier with Deep Learning

**Fig. 5:**
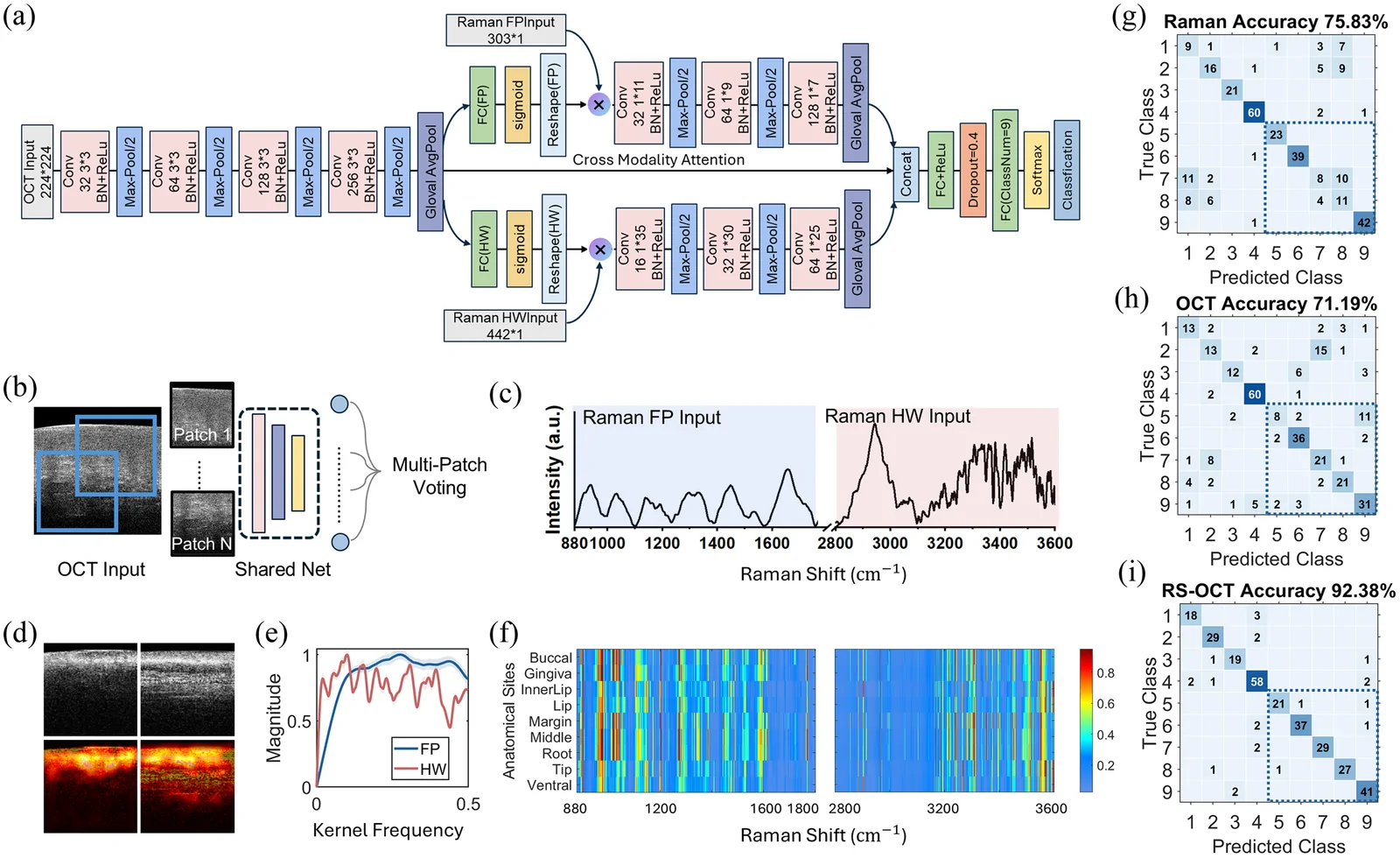
Architecture, interpretability, and diagnostic evaluation of the attention-gated network for morpho-chemical RS–OCT characterization. (a) Schematic of the proposed three-branch deep learning framework, integrating an OCT structural stream with dual-range Raman biochemical streams (FP and HW) coupled via a cross-modal attention (CMA) module. (b) Illustration of the patch-voting mechanism, where localized patches are randomly sampled from the OCT image and processed through a shared-weight 2D-CNN to extract robust texture-level morphological features. (c) Representative tissue Raman spectrum highlighting the segmented input regions for the fingerprint (FP, 880 to $1800\;{\mathrm{cm}}^{-1}$) and high-wavenumber (HW, 2800 to $3600\;{\mathrm{cm}}^{-1}$) branches. (d) Class activation mapping (CAM) of the OCT branch, visualizing the model’s focus on discriminative microstructural features such as epithelial stratification and basement membrane integrity. (e) Frequency response analysis of the first-layer Raman convolutional kernels, demonstrating the spectral specialization of the FP branch toward high-frequency molecular peaks and the HW branch toward low-frequency biochemical envelopes. (f) Heatmap showing the site-specific gating weights ($W_{\mathrm{fp}}$ and $W_{hw}$) generated by the CMA module, revealing adaptive spectral prioritization across the nine anatomical locations. (g)–(i) Confusion matrices for subject-independent fivefold cross-validation using Raman features alone (g), OCT patch voting alone (h), and the integrated RS–OCT fusion model (i). The class indices denote: (1) lip, (2) inner lip, (3) gingiva, (4) buccal, (5) root of tongue, (6) margin of tongue, (7) ventral tongue, (8) tip of tongue, and (9) middle of tongue.

Building on the co-registered *in vivo* dataset, the morpho-chemical deep learning model [Fig. 5(a)] demonstrated exceptional performance in the classification and discrimination of oral tissues. For the OCT branch, to mitigate the influence of global contours, the model introduced a shared-weight Patch Voting mechanism [Fig. 5(b)]. Validated through class activation mapping [CAM, Fig. 5(d)], this strategy successfully filtered out macro-geometric interferences, such as oral cavity shape and scanning angles, enabling the model to accurately lock onto biologically significant local morphological features, including epithelial thickness, basement membrane integrity, and lamina propria scattering intensity. Regarding the Raman branch, to avoid the mismatch in kernel width and unbalanced peak intensity associated with full-spectrum input, the model utilized convolutional kernels of varying sizes and counts for the two spectral regions [Fig. 5(c)]. Frequency response analysis [Fig. 5(e)] revealed that the fingerprint (FP) branch kernels exhibited stronger responses at higher spatial frequencies, capturing subtle molecular vibrational peak shifts, whereas the high-wavenumber (HW) branch focused on the low-frequency region to extract biochemical envelope features. This dual-branch physical isolation strategy compelled the model to spontaneously evolve differentiated feature extraction operators, effectively preventing the feature confusion prevalent in single-branch architectures handling full-spectrum data. This series of optimizations led to significant improvements in single-modality performance: Raman accuracy increased from 70.53% to 75.83% [Fig. 5(g)], whereas OCT accuracy surged from 56.29% to 71.19% [Fig. 5(h)].

The cross-modal attention (CMA) mechanism further demonstrated high biochemical specificity during the classification of nine oral anatomical sites [weight heatmap shown in Fig. 5(f)]. The model assigned significant weights to the 892 to $906\;{\mathrm{cm}}^{-1}$ region (C─O─C skeletal vibrations of the saccharide band), reflecting the active metabolic characteristics of mucosal tissues at the buccal, margin, and tip sites. In the 1025 to $1048\;{\mathrm{cm}}^{-1}$ band, the model exhibited extreme sensitivity to the Buccal category (weight up to 0.909), aligning closely with documented glycogen characteristic peaks and suggesting that abundant glycogen reserves in nonkeratinized buccal mucosa are a key identifying feature. The model identified the 1270 to $1340\;{\mathrm{cm}}^{-1}$ region (containing Amide III vibrations, lipid ${\mathrm{CH}}_{2}$ deformations, and DNA polynucleotide chain signals) as the core for cross-modal feature fusion, assigning very high weights (>0.9) across almost all categories. Finally, high weight distribution in the 1650 to $1680\;{\mathrm{cm}}^{-1}$ range (reaching 0.797 specifically for gingiva) precisely targeted Amide I structures and C═C stretching vibrations. By capturing differences in epithelial keratinization and stromal collagen content, the model established a logical closed loop between morphological structure recognition and specific biochemical monitoring. Near $2850\;{\mathrm{cm}}^{-1}$ (${\mathrm{CH}}_{2}$ symmetric stretching of fatty acids), gingiva (weight of 0.751), and margin exhibited high sensitivity, indicating the utilization of specific lipid distributions as identification markers. Near $2935\;{\mathrm{cm}}^{-1}$ (CH asymmetric stretching of proteins), the model reinforced features for middle (0.751) and inner lip, reflecting high-protein expression in these epithelial layers. Within the 3180 to $3250\;{\mathrm{cm}}^{-1}$ range, the model focused intensely on root, ventral, buccal, and middle (weights>0.83), proving that CMA successfully captured subtle variations in tissue hydration.

Ultimately, under fivefold cross-validation, the three-branch fusion model achieved an overall accuracy of 92.38%, with a fold-wise mean accuracy of 92.38%±0.92% [Fig. 5(i)]. The model successfully resolved the biochemical overlap of Raman spectra at the tongue tip and ventral tongue, where discrimination accuracies leaped from 37.93% and 25.81% to 93.10% (tip) and 93.55% (ventral), respectively. Specifically, in a targeted analysis of five high-risk cancer regions (i.e., tongue root, margin, ventral, tip, and middle), the average accuracy reached 93.37%, representing a significant improvement of 19.27% and 19.88% over Raman and OCT single-modalities, respectively. This cross-modal complementary mechanism—leveraging subtle anatomical layer differences captured by OCT (e.g., minor variations in papillary structures and muscle layer distribution) to compensate for the ambiguity of Raman biochemical features—demonstrates the model’s high robustness in fine anatomical localization. This high-precision localization capability provides a robust technical foundation for real-time lesion boundary demarcation and anatomical coordinate assistance in future clinical settings.

## 3 Discussion

In this study, we present the first realization of a single-fiber-enabled co-registered RS–OCT handheld probe capable of *in vivo* intraoral imaging. Unlike previous dual modal approaches that rely on bulky free-space optics or separate fiber bundles, which inherently suffer from co-registration errors and limited access to deep cavities,^19^^–^^24^ our system utilizes a single DCF to simultaneously deliver and collect RS-OCT signals from co-registered volume along the same optical axis. By packaging this architecture into a probe, we successfully demonstrated flexible access to multiple clinically relevant oral regions, including the anatomically challenging posterior tongue regions that are inaccessible to conventional probes. Crucially, this design ensures that high-resolution depth-resolved structural information and chemically specific fingerprints are acquired from a strictly co-registered tissue volume, providing a comprehensive biochemical–microstructural readout that is essential for accurate tissue characterization.

The synergistic value of this co-registered platform is evidenced by the distinct, yet complementary contrasts provided by the two modalities, which are effectively decoded and amplified by our deep learning framework. OCT is highly sensitive to the morphological heterogeneity across different oral anatomical sites: among the nine intraoral sites examined, the platform clearly captures differences in epithelial thickness, surface roughness, papillary and glandular structures, and the distribution of deeper muscle and connective tissues. Instead of relying on hand-crafted texture descriptors, the OCT patch-voting branch of our network automatically extracts these robust, texture-level morphological features, creating a structural context for the tissue. Meanwhile, Raman spectra directly report the biochemical composition, which is processed by a dual-branch network designed to capture both fingerprint and high-wavenumber signatures. Crucially, the strict spatial co-registration inherent to our single-fiber probe enables a cross-modality mechanism, which serves as a catalyst to fuse these streams. By allowing the structural embeddings from OCT to dynamically weight the biochemical features from Raman, the model effectively amplifies the discriminative power between class pairs that overlap in single-modality spaces (e.g., distinguishing the lip from the inner lip, or the tongue tip from the ventral tongue). This synergistic fusion led to a superior overall classification accuracy of 92.38%, representing a significant diagnostic gain over Raman (75.83%) and OCT (71.19%) single-modalities. These results indicate that the proposed platform can provide a reliable, anatomy-aware baseline for oral cavity tissue characterization, which is expected to be valuable for future oral cancer screening, lesion localization, and margin assessment.

The successful realization of this performance relies on a dedicated engineering strategy that resolves the historically conflicting optical requirements of RS and OCT. The DCF simultaneously satisfies the high-NA requirement for efficient Raman excitation and collection in the inner cladding and the lower-NA, larger-depth-of-focus needed for OCT imaging through the single-mode core, thereby overcoming the NA trade-off that has constrained conventional co-axial RS–OCT systems. However, using uncoated fiber for Raman delivery and collection inevitably introduces Raman background, originating from spontaneous Raman scattering within the glass itself. This background accumulates along the fiber propagation length and cannot be eliminated by conventional end-face bandpass and longpass filtering coatings, as such coatings are technically impractical to fabricate with the required precision at the micrometer-scale DCF core end face.^26^ Consequently, the only effective approach is to minimize the fiber length. Ultimately, to suppress fiber-generated Raman background, the DCF length is therefore intentionally minimized to 10.5 cm, which is incompatible with commonly used all-fiber DCF-coupler architectures in dual-modality systems.^28^^,^^29^ We therefore adopted a free-space coupling strategy, and in particular, implemented a cladding-mode-stripping protocol for the OCT channel to mitigate HOMs delivery and the mode-related artifacts it introduces, achieving a high FM excitation ratio of 92.21%. Nevertheless, backscattered HOMs-induced multipath artifacts remain unavoidable, and shortening the fiber even makes these artifacts fall closer to the true OCT signal (∼0.13  mm), as the separation is defined by the modal group-index difference multiplied by the fiber length. In this case, such artifacts cannot be pushed outside the imaging range as in other DCF-based OCT techniques.^27^^,^^28^ To strongly suppress these artifacts, we introduce a spatial aperture in the collimated beam path, effectively acting as an angular filter that preferentially transmits the FM while reducing HOM contributions by 5.62 dB. Together, this engineering solution including compact DCF optics, an optimized short-fiber design, free-space coupling, and spatial filtering of HOMs reconciles the physical constraints of the two modalities and enable high-performance dual-modal integration within a clinically compatible probe.

This DCF-based architecture offers distinct advantages over existing RS–OCT implementations. Compared with systems that rely on shared free-space objectives,^19^^–^^24^ our fiber-based approach avoids the inherent trade-off between the larger depth of field required for OCT and the high NA needed for efficient Raman collection and, importantly, enables a much more compact probe form factor, thereby making *in vivo* intraoral measurements feasible. Unlike dual-fiber-bundle or separate-path probes, where co-registration is achieved only through post-processing or complex alignment procedures, our single-fiber design guarantees intrinsic co-registration and is inherently robust against motion-induced misalignment during *in vivo* scanning. Furthermore, our spontaneous Raman-OCT probe maintains a footprint and cost profile, offering a practical balance between performance and deployability.

The clinical relevance of this platform lies in its ability to faithfully capture the natural heterogeneity of the oral mucosa *in vivo*. In this study, we established baseline structural-biochemical profiles across nine distinct anatomical sites, demonstrating the system’s high sensitivity to inter-site variations. Importantly, although only healthy volunteers were included, these site-specific “normal reference maps” provide an essential baseline framework for future lesion detection. Given the substantial intrinsic differences in epithelial thickness and biochemical composition across oral sites, applying a single global threshold or unified decision criterion would likely obscure subtle abnormalities and degrade diagnostic performance. By contrast, referencing each anatomical site to its own normal baseline is expected to substantially improve the sensitivity and specificity for early lesion detection. This capability is particularly crucial for the posterior tongue, which are considered susceptible regions for tongue and oropharyngeal squamous cell carcinoma and are difficult to assess using larger instruments due to their posterior anatomical location. The ability of our probe to perform real-time, noninvasive measurements in these posterior regions further enhances its potential for bedside diagnostics and rapid clinical decision support.

Despite these encouraging results, this study has several limitations that also point to directions for future work. First, although a Raman integration time of 0.5 s is relatively short and generally well tolerated by healthy volunteers, Raman measurements in patients are likely to be more susceptible to involuntary tongue motion and swallowing, which may degrade the Raman signal-to-noise ratio. Second, at this stage, we have only validated the system’s performance in distinguishing different anatomical sites using a relatively small cohort for proof of the RS-OCT technique; its diagnostic sensitivity and specificity for discriminating benign, premalignant, and malignant lesions, as well as a more rigorous assessment of statistical power, warrant further evaluation in larger, patient-based cohorts.

Correspondingly, future studies will require larger, multicenter cohorts spanning broader age ranges, high-risk groups, and patients with suspected or confirmed lesions to assess the robustness and generalizability of RS–OCT markers. Further optimization of laser power and detection efficiency, together with fast-readout or compressive spectrometer designs and deep-learning-based denoising and motion correction, could shorten acquisition times and further mitigate motion artifacts. Future probe iterations would incorporate a detachable side-view probe module to enable access to a broader range of oral subsites. Besides, more advanced multimodal attention and fusion frameworks tailored for early lesion detection, grading, and prognostication may better exploit the rich structural–biochemical information and further enhance the translational impact of the RS–OCT platform.

## 4 Conclusion

This study innovatively uses a single fiber to reconcile the optical design mismatch between RS and OCT while adopting a spatial filtering aperture to resolve the conflicts between OCT multipath artifact suppression and Raman fiber-background control, achieving co-registered single-fiber-enabled RS–OCT integration in a compact probe for the first time. With this platform, we obtained co-axial and co-registered structural and biochemical readouts across multiple intraoral sites in an *in vivo* setting and showed that multimodal fusion of RS-OCT diagnostic features with cross-modality deep learning markedly improves anatomical-site discrimination, providing a morpho-molecule foundation for enhancing *in vivo* oral lesion screening, lesion boundary assessment, and real-time clinical decision making in clinical settings.

## 5 Appendix: Methods and Materials

### 5.1 RS–OCT System Architecture

The hybrid RS–OCT platform was built around a single DCF-based handheld probe that provides co-axial delivery and collection channels for both modalities [Fig. 1(a)]. The system comprises a Raman excitation and detection module, an OCT imaging module, and integration optics that couple the two subsystems into the probe. For Raman spectroscopy, a 785-nm continuous-wave laser (I0785SR0090B-IS, Innovative Photonic Solutions) was used for excitation. The laser beam was filtered and focused into the inner-cladding channel of the DCF. The Raman excitation power at the tissue surface was maintained at ∼25.72  mW, which falls within the maximum permissible exposure limits for skin and mucosa defined by the ANSI Z136.1 standard. Backscattered Raman photons were collected by the same inner cladding, routed back through a dichroic combining and separating unit, and coupled into a fiber bundle connected to a CCD-based spectrograph for spectral acquisition. The spectrograph covered both FP and HW regions.

For OCT imaging, a 1300±70  nm SLD-based broadband light source operating at a 48 kHz A-line rate was used. The source output was launched into a fiber-based Michelson interferometer, where a 75/25 fiber coupler distributed light between the sample and reference arms, with the higher fraction directed to the sample arm to maximize detection sensitivity. In the DCF, the single-mode core guided both the illumination and backscattered signals, and the OCT sample-arm power at the probe tip was ∼2.35  mW, which is well within the established safety limits for 1300 nm skin and mucosa measurements. Polarization controllers were placed in the interferometer arms to compensate for birefringence-induced polarization mismatch, and the reference arm was dispersion-matched to the sample arm to reduce depth-dependent broadening of the axial PSF. The interference signal from the recombined sample and reference beams was directed to a complementary metal–oxidesemiconductor detector for spectrally encoded OCT signal acquisition.

The RS and OCT subsystems were integrated using a pair of dichroic mirrors ${\mathrm{DM}}_{1}$ (D102-R785, Semrock, Rochester, New York, United States) and ${\mathrm{DM}}_{2}$ (LP89661, Edmund Optics, Barrington, New Jersey, United States). ${\mathrm{DM}}_{1}$ combined the 785-nm Raman and 1300 nm OCT beams into a common path toward the DCF probe, whereas ${\mathrm{DM}}_{2}$ separated the returning Raman and OCT signals and directed them to their respective detection modules, ensuring that both structural and spectroscopic channels probed a co-registered tissue volume.

### 5.2 Single-Fiber-Based Probe Design

The 6-mm-outer diameter, 13.2-cm-front length handheld probe was based on a DCF and a miniature GRIN lens assembly packaged within a compact, rigid housing. The DCF (IXF-2CF-PAS-8-130-0.13, iXblue, Saint-Germain-en-Laye, France) featured a single-mode core (core diameter 8  μm, NA=0.13 at 1300 nm) surrounded by a multimode inner cladding (inner-cladding diameter 125  μm, NA=0.46 at 785 nm). The single-mode core was used exclusively for OCT illumination and signal collection, whereas the high–NA inner cladding provided an efficient channel for Raman excitation and collection, enabling both modalities to share a single, co-axial optical path. Proximal light coupling into the DCF was realized using a custom-engineered, high-precision free-space coupling assembly. The OCT source and Raman excitation beams were collimated by matched fiber collimators (F110APC-1310 for OCT and F110SMA-780 for Raman, Thorlabs Inc., Newton, New Jersey, United States) mounted on precision kinematic stages, allowing fine adjustment of lateral position (X, Y) and angular tip–tilt. To minimize back-reflections in OCT channel, both ends of the DCF were cleaved with an 8-deg angle. The OCT beam was first aligned to the DCF single-mode core. To experimentally realize and verify this core coupling, a systematic cladding-mode-stripping protocol was implemented: the DCF coating was partially removed by mechanical stripping, and index-matching oil was applied directly to the bare fiber while subjecting it to macro bending. This process effectively disrupted the cladding guidance, causing HOMs to leak out, thereby allowing us to adjust the collimator to maximize the pure core mode power. The oil was then removed and the fiber straightened to measure the total coupled power. Under these optimized conditions, the total OCT coupling efficiency reached 83.19%, with a measured FM power fraction of 92.21%, confirming high-purity forward propagation of the FM in good agreement with theoretical predictions. With the OCT coupling locked at its optimum and the co-axial beam geometry preserved, the Raman excitation beam was simultaneously coupled into the multimode inner cladding with an efficiency of ∼77.57%, ensuring sufficient excitation power at the distal fiber tip.

To limit fiber-generated Raman and fluorescence background while preserving sufficient flexibility and reach, the uncoated DCF segment inside the probe was restricted to a length of ∼10.5  cm. At the distal end of the DCF, a short GRIN lens was used to focus both the OCT and Raman beams and to define a common sampling volume. The GRIN element (diameter 1.8 mm, GT-LFRL-180-035, GRINTECH, Jena, Germany) was aligned coaxially with the DCF and fixed inside the probe tip using a precision-machined sleeve. The axial separation between the DCF end face and the GRIN lens was adjusted to yield a working distance on the order of 0.6 mm in tissue while maintaining the measured lateral resolution of ∼22  μm and axial resolution of ∼6.6  μm for OCT imaging. The same GRIN lens thus simultaneously provides an effective numerical aperture of ∼0.15 for the 1300 nm OCT core channel and ∼0.50 for the 785-nm Raman cladding channel, ensuring that the Raman and OCT channels interrogate a co-registered tissue volume.

All distal optics were enclosed in a stepped stainless-steel probe head, comprising a 6-mm-diameter distal front end and 10-mm- and 16-mm-diameter support sections, forming a rigid probe front end with an overall length of 132 mm. The tip of the tube was sealed with a flat fused-silica window to protect the GRIN lens and allow repeated disinfection for *in vivo* use. The internal components were potted with biocompatible, low-outgassing epoxy to enhance mechanical robustness and to minimize motion or misalignment during intraoral manipulation.

Lateral beam scanning was implemented by a piezoelectric tube (PZT) attached to the proximal end of the DCF within the probe handle. The PZT was driven by orthogonal voltage waveforms. During typical *in vivo* measurements, the PZT was driven at the resonance frequency of the 4.2-cm fiber cantilever (∼57  Hz), generating an OCT lateral scanning field of ∼1.6  mm at the sample.

### 5.3 Multipath Artifact Suppression in OCT Images

To suppress multipath artifacts arising from HOMs in OCT path, we implemented a spatial filtering aperture in the OCT relay optics. Notably, the iris is inserted in the OCT detection path to filter HOMs and therefore does not affect OCT core excitation or the Raman excitation/collection channel. An adjustable iris diaphragm was placed at an intermediate image plane in the common-path relay between the DCF–GRIN probe and the detection fiber [Fig. 3(c)], where it blocked peripheral rays and preferentially transmitted the central portion of the beam. By truncating the outer beam regions that are predominantly carried by HOMs, the iris reduced their contribution to the detected OCT signal while largely preserving the FM ($LP_{01}$) in the core. In addition to decreasing HOMs intensity, the aperture effectively reduced the beam diameter and working numerical aperture, slightly enlarging the focal spot and further decreasing the backward coupling efficiency of HOMs into the single-mode detection fiber.

To quantitatively optimize the aperture diameter, we performed numerical simulations of beam propagation for the FM and the dominant core-like HOMs supported by the DCF. Among these HOMs, the two strongest modes exhibited initial intensities of 25.07% and 19.34% relative to the FM at the fiber output (see Sec. S4 in the Supplementary Material), whereas the other HOMs intensity are negligible. The field profiles of each mode were propagated with different iris diameters, and the focal-plane intensity distributions and on-axis coupling efficiencies were calculated. The simulated focal spot profiles with and without the iris highlight that the aperture induces more pronounced power loss and spot-size broadening for HOMs than for FM. The resulting intensity ratios as a function of aperture diameter showed that decreasing the iris diameter strongly attenuates HOMs while causing only modest loss for the FM. Based on this trade-off, we selected an iris diameter of 0.9 mm, for which the FM attenuation is <2  dB, whereas the attenuation of the two dominant HOMs exceeds 5 dB.

The simulated trends were validated experimentally by measuring the OCT point-spread function (PSF) with and without the spatial filtering aperture. PSFs were acquired by placing a reflective mirror at the focal plane, averaging multiple A-scans, and analyzing the main peak and the delayed secondary peak associated with HOMs-related multipath artifacts. Without the iris, the HOMs peak was ∼11.34  dB below the FM, whereas with the 0.9-mm aperture, the HOMs peak was reduced to ∼16.96  dB below the FM, in good agreement with the simulations. *In vivo* fingertip OCT B-scans further confirmed that the optimized aperture markedly suppresses the ghost interface generated by multipath artifacts and separates it from the true tissue surface.

To further minimize the impact of residual multipath artifacts, we implemented an automatic surface detection on OCT B-scans using a deep-learning-based workflow. First, it preprocesses the B-scans to extract edge information and trains a U-Net segmentation model to identify the upper tissue region.^32^ Then, for surface reconstruction, it applies a constrained random sampling strategy to select reliable surface points that satisfy strict distance and smoothness criteria, followed by cubic spline interpolation to generate a smooth and accurate surface profile. Finally, all voxels located above this surface were set to zero, retaining only subsurface contents and effectively removing residual above-surface multipath artifacts.

### 5.4 *In Vivo* Co-Registered Intraoral Measurements

*In vivo* intraoral RS–OCT measurements were conducted in 15 healthy adult volunteers (nine males and six females, aged 19 to 43 years). For each volunteer, measurements were performed at nine anatomically distinct intraoral sites: lip, inner lip, gingiva, buccal mucosa, root of tongue, margin of tongue, ventral tongue, tip of tongue, and middle of tongue, as illustrated in Fig. 4(a). During data acquisition, the oral cavity was gently opened to expose the target site, and the flat fused-silica window at the distal end of the probe was gently placed against the mucosal surface to maintain a stable and approximately perpendicular probe–tissue interface, thereby ensuring reliable acquisition of both OCT images and Raman spectra. Between participants and between different sites, the probe window was cleaned and disinfected according to standard clinical hygiene procedures.

OCT and Raman data were acquired sequentially from the same measurement site. A rapid large-area OCT scan was first performed to localize and center the region of interest within the scan field. The Raman spectrum was then acquired with the fiber returned to its rest position, which coincides with the center of the OCT scan field. For OCT, B-scans were acquired at an A-line rate of 48 kHz using the DCF core channel, with a lateral scan range of 1.6 mm and a depth range ∼1.2  mm. A sufficient number of B-scans were recorded at each site to ensure stable image quality; for subsequent analysis, three to five representative frames with minimal motion artifacts were selected.

Immediately following OCT imaging at the same site, a co-registered Raman spectrum was acquired using the DCF inner-cladding channel. At each location, three to five repeated Raman acquisitions were recorded to improve signal-to-noise ratio and assess repeatability; spectra contaminated by motion or poor contact (with the $2935\;{\mathrm{cm}}^{-1}$ peak falling below the noise level) were excluded during preprocessing. The criterion led to the exclusion of only one measurement, corresponding to a root of tongue site in one volunteer. This protocol yielded a co-registered dataset of OCT B-scans and Raman spectra across nine intraoral sites in each volunteer, providing paired structural and biochemical information for subsequent feature extraction and multisite tissue classification.

### 5.5 Data Preprocessing

Raw spectral-domain OCT data from the Telesto 330 engine were first reconstructed using the vendor software, which performs background subtraction, k-space resampling, numerical dispersion compensation, and fast Fourier transform to generate calibrated depth-resolved A-lines. Within the co-registration volume, voxel intensities were linearly normalized to the [0, 255] gray-level range. Because the lateral PZT scanner follows a sinusoidal trajectory, the raw B-scans exhibit nonuniform lateral sampling density. To correct this distortion, the sinusoidal scan was linearized by mapping each half-cycle of the PZT waveform onto a uniformly spaced lateral grid (sine-scan correction), effectively restoring constant pixel spacing across the B-scan.

The raw Raman spectra undergo several processing steps, including smoothing, background removal, normalization, and principal component feature extraction. First, a Savitzky–Golay filter is applied to smooth the spectra and reduce shot noise. The filter window width is set to 5 pixels for the fingerprint (FP) region (800 to $1800\;{\mathrm{cm}}^{-1}$) and 7 pixels for the high-wavenumber (HW) region (2800 to $3600\;{\mathrm{cm}}^{-1}$), aligning with the spectral resolution of the fiberoptic Raman spectroscopy system. Next, the tissue autofluorescence background is removed using an iterative polynomial fitting approach with a fifth-order polynomial for the FP region and a first-order polynomial for the HW region. ^6^^–^^8^ After normalization, the three to five spectra collected at each site are averaged, which is carried forward for classification.

### 5.6 Cross-Modality Deep Learning Network

To fully exploit the synergistic potential of morphological features from OCT and biochemical signatures from Raman, we developed a three-branch deep learning fusion model incorporating OCT patch voting, dual-region Raman input, and a cross-modal attention mechanism. In the OCT structural branch, a shared-weight patch-voting strategy was implemented to reduce the model’s reliance on global contours and compel the network to extract robust, texture-level morphological features. Original OCT images were normalized and resized to 288×288  pixels. During the training phase, local patches of 224×224 were randomly cropped from the original images. For each image, nine patches were extracted and fed into a shared-weight 2D-convolutional neural network (CNN), with final classification decided by averaging the Softmax probability scores of these patches. The OCT branch consists of four consecutive feature extraction blocks with a uniform convolutional kernel size of 3×3, whereas the number of channels increases from 32 to 64, 128, and 256. Each convolutional block is followed by batch normalization and ReLU activation, with 2×2  max-pooling layers appended after the first three blocks. Finally, a global average pooling (GAP) layer compresses the high-dimensional feature maps into a 256-dimensional structural embedding vector, *Voct*.

The cross-modal attention module utilizes *Voct* as a guidance signal to adaptively weight the raw Raman spectra. *Voct* is fed into two independent gating subnetworks: the FP gating layer employs a fully connected layer to map 256 to 303 dimensions followed by a Sigmoid activation to generate a weight vector Wfp, consistent with the FP spectral dimensions; the HW gating layer maps 256 to 442 dimensions to generate the weight vector Whw. These weight vectors are element-wise multiplied with the input raw spectra, achieving a data-driven integration of morphological context and specific molecular signatures. The Raman branches receive the gated spectra and further extract deep biochemical features using 1D-CNNs. The FP and HW sub-branches utilize three convolutional layers with kernel sizes of [11, 9, 7] and [35, 30, 25], respectively, with channel configurations of [32, 64, 128] and [16, 32, 64]. Both sub-branches incorporate 1D max-pooling layers (stride 2) between convolutions and employ GAP layers to derive their respective biochemical feature vectors. The model fuses the OCT vector (256D), FP vector (128D), and HW vector (64D) through concatenation. The combined vector is processed by a 256D fully connected layer, followed by ReLU activation and a dropout layer (rate=0.3) to mitigate overfitting. Final prediction probabilities for the nine tissue categories are generated via a Softmax layer.

The model was trained using the Adam optimizer with a learning rate of $6\times 10^{-4}$ and a L2 regularization coefficient of $1\times 10^{-4}$ for 200 epochs. A subject-level five-fold cross-validation strategy was employed across the recruited volunteers. The dataset was partitioned by subject identity into training, validation, and testing subsets following a 3:1:1 ratio, ensuring that data from any single participant were never shared across different subsets. Ultimately, this cross-modality architecture and rigorous evaluation framework ensures a synergistic fusion of morphological and biochemical features while maintaining high clinical generalizability.

## Supplementary Material

10.1117/1.BIOS.3.3.035004.s01

## Data Availability

Data underlying the results presented in this paper are not publicly available at this time but may be obtained from the corresponding author upon reasonable request.
