# Supplementary material for: Single-fiber co-registered Raman-OCT technique with deep learning enhances in vivo oral tissue assessment
Source: Biophotonics Discov. 2026 Aug 27;3(3):035004. doi: 10.1117/1.BIOS.3.3.035004 (PMC13557451; doi:10.1117/1.BIOS.3.3.035004)
Supplement: Supplementary file 1 [file BIOS_003_035004_SD001.pdf]

Supporting Information for

Single-Fiber Co-registered Raman-OCT Technique with Deep Learning Enhances In Vivo Oral Tissue Assessment

Chang Liu[1], Bin He[1], Kan Lin[1], Chi Shu[1], Wei Zheng[1], Zhiwei Huang[1,2,3,4*]

[1]Optical Bioimaging Laboratory, Department of Biomedical Engineering, College of Design and Engineering, National University of Singapore, Singapore 117576

[2]National University of Singapore (Suzhou) Research Institute, Suzhou, Jiangsu 215123, China

[3]National University of Singapore Guangzhou Research Translation and Innovation Institute, Guangzhou, Guangdong 510700, China

[4]NUS Graduate School for Integrative Sciences and Engineering Programme (ISEP), National University of Singapore, Singapore 119077

[*] **Correspondence to:**

Dr. Zhiwei Huang, Optical Bioimaging Laboratory, Department of Biomedical Engineering, College of Design and Engineering, National University of Singapore, 9 Engineering Drive 1, Singapore 117576

Tel: +65- 6516-8856

Fax: +65- 6872-3069

E-mail: biehzw@nus.edu.sg

S1. Coupling Efficiency in Forward Propagation of DCF-GRIN RS-OCT technique

The coupling efficiency of fundamental mode (FM, LP_{01}) and higher-order modes (HOMs, e.g., modes LP_{11}, LP_{21}, LP_{02}, LP_{12} and LP_{22}) are calculated based on the overlap between the beam spot profile and the mode distribution within the DCF.

$$\eta = |\iint E_1(x,y) * E_2(x,y)\, dxdy|^2 / [\iint |E_1(x,y)|^2\, dxdy * \iint |E_2(x,y)|^2\, dxdy]. \quad \text{(S1)}$$

where $E_1(x,y)$ represents the mode distribution of the DCF, and $E_2(x,y)$ is the incident light beam. The numerator in Eq. S1 accounts for the overlapping portion of the modes (indicating the degree of mode matching), while the denominator ensures normalization. For example, in the forward coupling procedure in DCF-GRIN probe, E_1 is the fiber mode profile obtained from COMSOL simulations. E_2 represents the focal intensity with a Gaussian beam profile formed by focusing the single-mode fiber (SMF) output through a pair of lenses, and is given by:

$$E_2(x,y) = E_0 * \exp\left(-\frac{x^2+y^2}{w_0^2}\right) \quad \text{(S2)}$$

where w_0 is the beam waist. The calculated coupling efficiency for the different modes are: 82.23% (LP_{01}, FM), 2.37% (LP_{02}), 0.002% (LP_{21}) and 0.0003% (LP_{22}).

S2. GRIN-DCF Configuration Parameters

For the DCF-GRIN probe design, the gap between DCF and GRIN lens is a key parameter which controls the effective NA of DCF-GRIN probe in optimizing the excitation/collection efficiency of OCT and RS signals. The performance metrics are determined by the effective NA values: Raman spectra SNR is proportional to the square of the RS collection NA. For OCT, the lateral resolution is inversely proportional to the NA, while the depth of focus is inversely proportional to the square of the NA. To quantitatively evaluate the effective NA changes from varying the GRIN-DCF distance in our probe design, simulations are conducted using ZEMAX and MATLAB. With the increase of GRIN-DCF distance, the working distance would decrease, and the effective NA for RS and OCT would increase correspondingly, resulting in an

enhanced Raman signal collection, improved OCT lateral resolution, but a reduced depth of focus. Considering these performance parameters, a 1.2 mm GRIN-DCF distance is chosen to optimize the collection efficiency, such that the RS collection NA reaches the GRIN lens limit. In this configuration, the RS and OCT working distance is 0.6 mm, the effective NAs of RS and OCT modalities are 0.5 and 0.15, respectively.

S3. Monte Carlo Simulations and Depth Validation of Raman Probing Volume

We developed a MC simulation model to study Raman light generation and propagation in epithelium-stroma tissue as well as Raman signal collection with the use of GRIN lens in DCF-GRIN probe design. The modified MC codes include the following modules: (i) illumination light propagation in GRIN lens and tissue; (ii) Raman photons generation and propagation in tissue; (iii) Raman photons collection by the GRIN lens and propagation in the GRIN lens; (iv) Raman photons collection by the DCF.

In MC simulations, ten million illumination light photons are launched into the tissue through the GRIN lens. The launch radius is defined by the inner cladding diameter of the DCF, and the launch angles range from 0 to 0.2 sr as governed by the NA of the excitation Raman light. The center of the DCF distal end serves as the origin for both the depth and radius coordinates, with the rightward direction along the radius and upward along the depth axis defined as positive.

In each movement of photon, the position and direction are updated based on its current trajectory, step size, and angle relative to the optical axis (θ). The equations governing the propagation are:

$$d_{i+1} = d_i + step \cdot cos\theta \quad \text{(S3)}$$

$$r_{i+1} = r_i + step \cdot sin\theta \quad \text{(S4)}$$

where d_{i+1} and r_{i+1} are the updated axial and radial positions, respectively. θ remains the same when light transmits in air while at the media interface, GRIN lens and tissue would change the angle. Refraction at interface obeys Snell's law influenced by refraction index of two media. Continuous angle change in GRIN lens is due to the

refractive index gradient in infinitesimal regions. The deflection angle (θ) of each scattering in tissue is characterized by Henyey-Greenstein (H-G) phase function:

$$P(cos\theta) = \frac{1}{4\pi} \cdot \frac{1-g^2}{(1+g^2-2gcos\theta)^{3/2}} \quad \text{(S5)}$$

where g is the anisotropy factor, which determines the scattering directionality. A two-layer epithelium-stroma model is utilized to study the propagation of illumination light and Raman photons within the tissue. Referred to the bio-samples used in the study, the epithelium thickness is set to be 200 μm. For illumination wavelength (785 nm), the scattering coefficient (μ_s), absorption coefficient (μ_a), anisotropy factor (g) and refractive index (n) are 20 cm^{-1}, 0.8 cm^{-1}, 0.9 and 1.4 for the epithelium, and 120 cm^{-1}, 0.3 cm^{-1}, 0.9 and 1.4 for the stroma, respectively. For Raman scattered wavelength (820 nm), μ_s, μ_a, g and n are set to 19 cm^{-1}, 0.7 cm^{-1}, 0.8 and 1.4 for the epithelium, and 115 cm^{-1}, 0.28 cm^{-1}, 0.8 and 1.4 for the stroma, respectively. [1,2]

The generation of Raman scattering is quantified by the probability (p) of the illumination light being converted into Raman scattered light, expressed as:

$$p = \frac{\mu_a}{\mu_a+\mu_s} \cdot RY \quad \text{(S6)}$$

where μ_a is the absorption coefficient for generating Raman photons, μ_s is the scattering coefficient at Raman scattered wavelength, and RY is the Raman quantum yield. Back-scattered Raman photons which pass through the GRIN lens within the DCF inner cladding collection core (NA = 0.46) will be coupled into the DCF and be considered as the collected Raman photons by the hybrid RS-OCT probe as shown in Figure 2(d).

We further performed an experimental characterization of the axial Raman response provides additional validation of the sampling volume inferred from the Monte Carlo simulation. A silicon wafer was used as a spatially localized Raman reporter. To approximate measurements under scattering conditions, the silicon wafer was first coated with a 100-μm-thick lipid layer, which was subsequently covered with a 30-μm-thick polymer film. Starting from the nominal working-distance position, defined as $z = 0$, the sample was translated axially away from the probe, and the integrated area of the silicon Raman band at approximately 520 cm^{-1} was calculated at

each axial position after baseline correction (Figure S1(a)). As shown in Figure S1(b), the silicon Raman signal decreased to half of its maximum value at an axial displacement of approximately 370 µm, while the characteristic silicon band at 520 cm^{-1} remained detectable up to an axial displacement of approximately 1000 µm. The experimentally observed interrogation range is consistent with the region predicted by the simulation and therefore supports the interpretation of the simulated vertical interrogation depth and its overlap with the OCT imaging region.

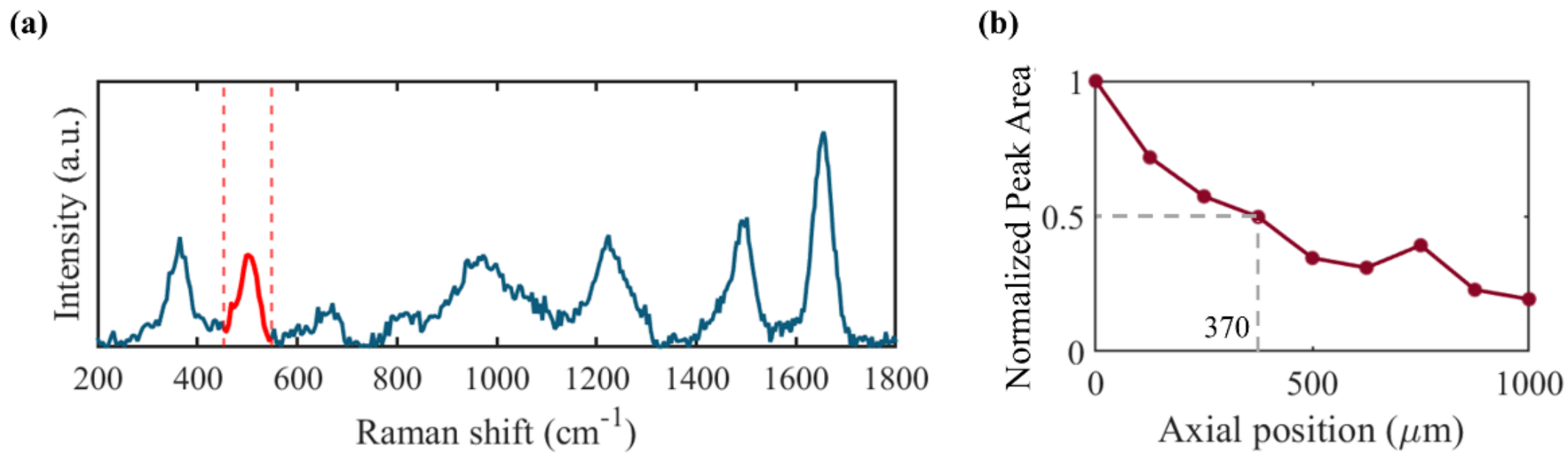

Figure S1. Experimental characterization of the axial Raman interrogation response. (a) Representative Raman spectrum acquired from a silicon wafer covered with a 100-µm-thick lipid layer and a 30-µm-thick PP film. The silicon Raman band at approximately 520 cm^{-1} and the spectral range used for peak-area integration are highlighted in red. (b) Normalized peak area of the silicon Raman band as a function of axial displacement from the working-distance position.

S4. Intensity ratio of HOMs to FM

In the back-scattered coupling process, to determine the intensity ratio of different modes coupled into the DCF, we approximate each mode as a channel characterized by a specific transverse position and acceptance angle. We then apply the extended Huygens-Fresnel (HF) model to compute the OCT signal distribution and simulate the collected intensity for different mode channels. [3] The HF model is expressed:

$$\Psi(z, s_0, \alpha) = e^{-2\mu_a z}\left[e^{-2\mu_s z} e^{-\frac{(z\alpha+s^0)^2}{2w_H^2}} + \frac{4e^{-2\mu_s z}(1-e^{-2\mu_s z})}{(1+\mu_a \Delta z_D)\left(1+\frac{w_{SA}^2}{w_H^2}\right)} + \frac{(1-e^{-\mu_s z})^2 w_H^2}{(1+\mu_a \Delta z_D) w_{SA}^2} e^{-\frac{(z\alpha+s^0)^2}{2w_{SA}^2}}\right]$$

(S7)

where $\Psi(z, s_0, \alpha)$ is the Heterodyne efficiency factor, representing the intensity of the OCT signal. μ_a and μ_s are the absorption coefficient and scattering coefficient, respectively. ω_H is $1/e^2$ intensity radius in the absence of scattering and absorption. ω_{SA} is $1/e^2$ intensity radius considering both scattering and absorption. s_0 is initial lateral offset and α is an angular offset. z is OCT imaging depth and Δz_D is the depth correction factor related to scattering and absorption. We use transverse positions of 0, 6, 7, 13, 18 and 23 μm, along with acceptance angles of 0, 0.08, 0.09, 0.17, 0.19, and 0.2 sr, to represent the LP_{01} (FM), LP_{11}, LP_{21}, LP_{02}, LP_{12}, and LP_{22} modes, respectively.

Figure 3(d) illustrates the OCT intensity distribution of different modes along the depth. By integrating the signal over the 0-1 mm range, we obtain the intensity ratios of back-scattered HOMs to the FM as: 47.88% (LP_{11}), 38.41% (LP_{21}), 3.74% (LP_{02}), 3.19% (LP_{12}) and 4.21% (LP_{22}). Thus, the HO multi-pass artificial contributions to OCT imaging decrease successively, with major artifacts arising from LP_{11} and LP_{21}modes.

S5. Suppression of higher-order modes of back-scattered light in DCF-GRIN OCT tissue imaging

S5.1 Estimations of coupling efficiency of the back-scattered light with different HOMs

The back-scattered light emitted from the DCF follows a Laguerre-Gaussian (LG) distribution. Based on the angular distribution and radial node characteristics, the approximate relationship between LP modes in optical fibers and LG modes can be summarized as follows: The FM (LP_{01}) approximately corresponds to LG_{00}; For HOMs, LP_{11} corresponds to LG_{01}, LP_{21} corresponds to LG_{02}, LP_{02} corresponds to LG_{10}, LP_{12} corresponds to LG_{11}, and LP_{22} corresponds to LG_{12}. The LG distribution is expressed as:

$$E_{m,n}(r, \phi, z) = \frac{1}{w(z)} * \left(\frac{\sqrt{2}r}{w(z)}\right)^{|n|} * L_m^{|l|}\left(\frac{2r^2}{w^2(z)}\right) * \exp\left(\frac{-r^2}{w^2(z)}\right) * \exp\left(ik\frac{r^2}{2R(z)}\right)$$

$$* \ exp(-i(2m+|n|+1)\zeta(z)) \ * \ exp(il\phi) \quad \text{(S8)}$$

where m and n are the radial mode number and the angular momentum mode number, respectively. $w(z)$ represents the beam radius at position z, $R(z)$ is the wavefront curvature radius, and $\zeta(z)$ is the Gouy phase, which describes the phase shift as the beam propagates. These properties can be referred to Gaussian beam.

The focusing process of a Laguerre-Gaussian beam through a lens system can be described by Fourier transform analysis. The electric field distribution at the focal point is given by:

$$E_{focus}(r,\phi) = \mathcal{F}\{E_{LG}(r,\phi)\} \sim (\frac{\sqrt{2}r}{w_0})^{|n|} * L_m^{|l|}(\frac{2r^2}{w_0^2}) * exp(\frac{-r^2}{w_0^2}) * exp(il\phi) \quad \text{(S9)}$$

The coupling efficiency for different high-order modes of back-scattered light can be calculated based on Eq. S1, where $E_2(x,y)$ is taken as the back-scattered beam profile as represented by Eq. S9, and $E_1(x,y)$ is the Gaussian modal distribution of the collection SMF as described by Eq. S2, but the waist w_0 is defined by collection SMF in DCF. Hence, FM and the dominant HOMs, LP_{11} and LP_{21} coupling efficiencies are 81.16%, 50.67% and 44.71%, respectively. Multiplied the HOMs intensity calculated in Section S4, the final mode intensity ratio to FM are 24.25% (LP_{11}) and 21.16% (LP_{21}), which are the main HOMs needed to be suppressed.

S5.2 Suppression of HOMs-induced-artifacts in OCT images

The HOMs would create multipath artifacts in OCT imaging. Meanwhile, LP_{11}, LP_{21} and LP_{02} exhibit considerable intensity ratios relative to the FM. Mitigating HOM images should be considered to improve the OCT image quality. We take advantage of the free space optics in our DCF-GRIN probe design and employ an iris to restrict the beam diameter of the backscattered light. The introduction of a varying iris aperture affects the DCF-GRIN system by modulating the LG beam $E_{LG}(r,\phi)$ with the aperture function $A(r,\phi)$. This modulation effectively filters out part of the light intensity at higher scattering angles. The resulting electric field distribution can be expressed as:

$$A(r,\phi) = \begin{cases} 1, r < R \\ 0, r \geq R \end{cases} \tag{S10}$$

$$E_{focus\_filtered}(r,\phi) = \mathcal{F}\{E_{LG}(r,\phi) * A(r,\phi)\} \tag{S11}$$

where R is the iris radius. While still maintaining most power of FM, the iris diaphragm significantly suppresses the interferences of back-scattered HOMs. LP_{11} and LP_{21} with higher intensities are the main HOMs that need to be more effectively suppressed. Figure S2(a) shows remaining power ratios of HOMs to FM across varying iris radii. The iris diaphragm blocks peripheral light, suppressing HOMs while largely preserving the FM. Moreover, the reduced beam diameter and NA lead to an enlarged spot size, which further decreases the backward coupling efficiency of HOMs, as shown in Figure S2(b). The mode intensity, as the product of coupling efficiency and remaining power ratio, along the iris radius are displayed in Figure 3(e) in the main text, demonstrating the effective reduction of the absolute intensity of back scattered HOMs with respect to FM through the implementation of iris diaphragm, leading to a significant suppression of multipath artifacts in the OCT signal.

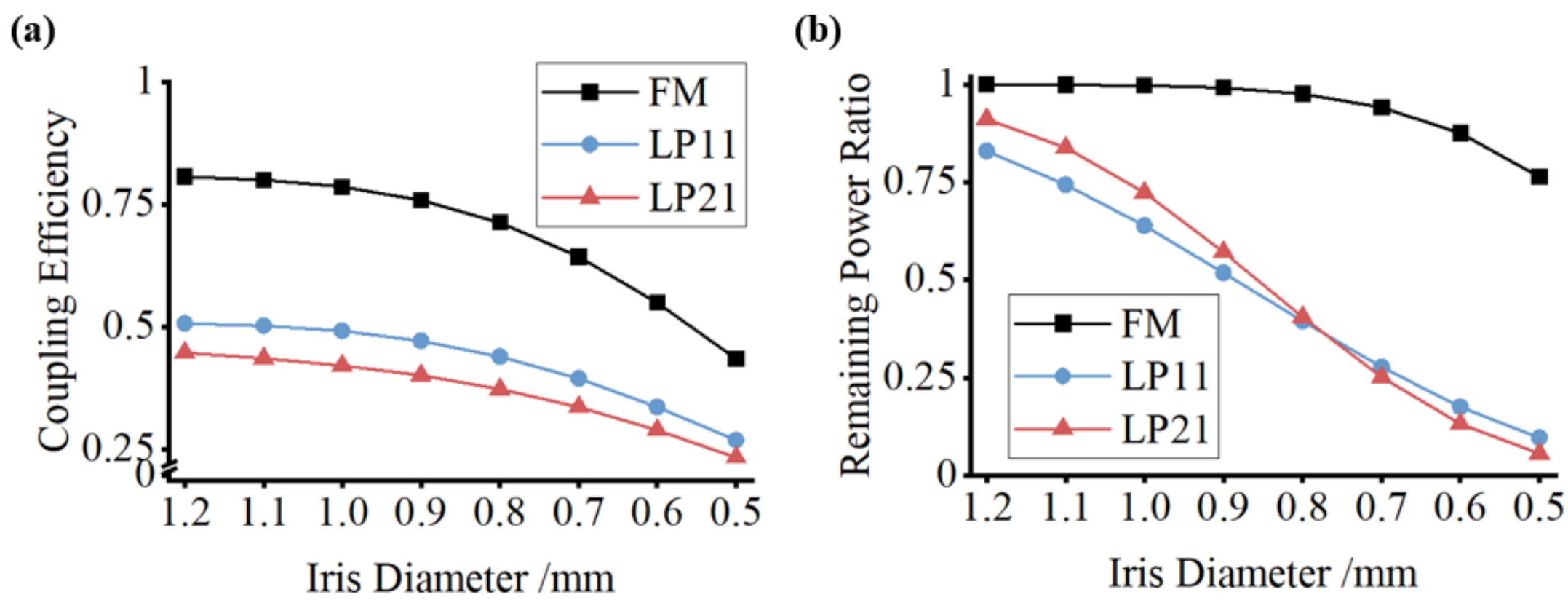

Figure S2. Effect of the iris diaphragm on backward coupling efficiency and remaining power ratio in the OCT subsystem. (a) Coupling efficiency of the back-scattered light with different modes across varying iris radius. (b) Remaining power ratios of different modes across varying iris radius.

References

1. Chang, S. K., Arifler, D., Drezek, R., Follen, M. & Richards-Kortum, R. Analytical model to describe fluorescence spectra of normal and preneoplastic epithelial tissue: comparison with Monte Carlo simulations and clinical measurements. *J Biomed Opt* **9,** 511–522 (2004).
2. Zaane, F. van L., Gamm, U. A., Driel, P. B. A. A. van, Snoeks, T. J. A., Bruijn, H. S. de, Heuvel, A. van der P. den, Mol, I. M., Löwik, C. W. G. M., Sterenborg, H. J. C. M., Amelink, A. & Robinson, D. J. In vivo quantification of the scattering properties of tissue using multi-diameter single fiber reflectance spectroscopy. *Biomed. Opt. Express, BOE* **4,** 696–708 (2013).
3. Untracht, G. R., Chen, M., Wijesinghe, P., Mas, J., Yura, H. T., Marti, D., Andersen, P. E. & Dholakia, K. Spatially offset optical coherence tomography: Leveraging multiple scattering for high-contrast imaging at depth in turbid media. *Sci. Adv.* **9,** eadh5435 (2023).
4. Agrawal, A., Pfefer, T. J., Woolliams, P. D., Tomlins, P. H. & Nehmetallah, G. Methods to assess sensitivity of optical coherence tomography systems. Biomed Opt Express 8, 902–917 (2017).
